# Chitosan-Coated Magnetic Nanoparticles Prepared in One-Step by Precipitation in a High-Aqueous Phase Content Reverse Microemulsion

**DOI:** 10.3390/molecules19079273

**Published:** 2014-07-02

**Authors:** María Guadalupe Pineda, Silvia Torres, Luis Valencia López, Francisco Javier Enríquez-Medrano, Ramón Díaz de León, Salvador Fernández, Hened Saade, Raúl Guillermo López

**Affiliations:** Departamento de Procesos de Polimerización, Centro de Investigación en Química Aplicada, Blvd. Enrique Reyna No. 140, C.P. 25294 Saltillo, Coahuila, Mexico

**Keywords:** chitosan magnetic nanoparticles one-step, heavy metal ion removal, high productivity microemulsion precipitation

## Abstract

Chitosan-coated magnetic nanoparticles (CMNP) were prepared in one-step by precipitation in a high-aqueous phase content reverse microemulsion in the presence of chitosan. The high-aqueous phase concentration led to productivities close to 0.49 g CMNP/100 g microemulsion; much higher than those characteristic of precipitation in reverse microemulsions for preparing magnetic nanoparticles. The obtained nanoparticles present a narrow particle size distribution with an average diameter of 4.5 nm; appearing to be formed of a single crystallite; furthermore they present superparamagnetism and high magnetization values; close to 49 emu/g. Characterization of CMNP suggests that chitosan is present as a non-homogeneous very thin layer; which explains the slight reduction in the magnetization value of CMNP in comparison with that of uncoated magnetic nanoparticles. The prepared nanoparticles show high heavy ion removal capability; as demonstrated by their use in the treatment of Pb^2+^ aqueous solutions; from which lead ions were completely removed within 10 min.

## 1. Introduction

Magnetic nanoparticles (MNP) constitute a very interesting material due to their wide range of applications in the technological [1] and biomedical fields [2]. Technological uses include density magnetic recording media, sensors, catalysts [3] and contaminant removal [4,5], among others. On the other hand, biomedical uses mainly include diagnosis [6,7,8], drug delivery [2,8] and in hyperthermia [2,9]. In most of the MNP applications is required that they be covered by a layer of material, such as a polymer, to increase their stability [2,10]. In addition to protection, in certain medical applications the MNP coating is used with purposes such as modification of the surface charge of the nanoparticles, reduction of immunogenicity risk, increase of cellular uptake [11] and surface functionalization [2]. In the latter applications, chitosan is extensively used as material for MNP covering, due to its biodegradability, non-toxicity, hydrophilicity, and the easiness with which the amino and hydroxyl groups on its surface can be used to further functionalize the particles, diversifying enormously their use possibilities [12].

The most common method for preparing chitosan-coated magnetic nanoparticles (CMNPs) is by first obtaining MNP using a coprecipitation method, followed by recovery of the nanoparticles and then covering them with chitosan in a further step [13,14]. However, some works have recently documented a one-step CMNP preparation process, which relies on the coprecipitation of nanoparticles in the presence of chitosan [15,16,17,18]. The first documented efforts on this subject [15,16] gave rise to CMNPs with relatively large diameters (>50 nm) and median values of saturation magnetization (≈35 emu/g), which can be ascribed to the high chitosan content in the coated nanoparticles (>30 wt%). Nanoparticles of smaller sizes with a broad range of saturation magnetization values have been reported in subsequent works. This way CMNPs slightly larger than 10 nm in average diameter were prepared by Liu *et al.* [19], Wang *et al.* [20] and Li *et al.* [21] and up to 40 nm by Yuwei *et al.* [4], albeit with large differences in saturation magnetization: 13.4 [9], 36 [4] and 55 emu/g [20], while Li *et al.* did not report any magnetization value. On the other hand, the work of Unsoy *et al.* [11] is noteworthy because they obtained very small CMNPs (<5 nm in average diameter), although the saturation magnetization value in this case was only around 25 emu/g. Our group has also published some reports on the preparation of CMNPs in one step by coprecipitation in the presence of chitosan [17,18]. Our results mainly show that is possible to obtain small CMNPs with a very small chitosan content, which only slightly reduces the magnetization, as compared to that of the naked magnetic nanoparticles. In fact, the very small size (≈10 nm in average diameter) endows them with superparamagnetic character; in addition, the CMNPs showed a relatively high final magnetization value of 65.6 emu/g at room temperature and 20 kOe.

More recently, our group reported the use of reverse microemulsions as templates for obtaining CMNPs in one-step [22]. As far as we know this would be the first report in the specialized literature on the use of this method for preparing this type of nanoparticles. Microemulsions are thermodynamically stable systems composed of an aqueous phase, an oil or organic phase and one or more surfactants [23]. In the case of reverse microemulsions the system consists of reverse micelles swollen with the aqueous phase and typically with mean diameters < 10 nm, dispersed in the oil phase. These nanostructures have been used for some time now as templates for preparing metal and metal oxide nanoparticles, including some with magnetic properties [24].

In our last work on the one-step preparation of CMNP, we reported the use of a reverse microemulsion composed of 0.25 M aqueous solution of a FeCl_3_·6H_2_O/FeCl_2_·4H_2_O mixture (3/2, mol/mol) containing 0.1 wt% chitosan, toluene as organic phase and a mixture of sodium bis(2-ethylhexyl) sulfosuccinate (AOT) and sodium dodecyl sulfate (SDS) (2/1, w/w) as surfactant [22]. In that instance, a relatively low aqueous phase concentration (15 wt%) was employed. Here in we report a one-step CMNP preparation in a reverse microemulsion with much higher aqueous phase content (26 wt%), which notably increases the process productivity of nanoparticles covered with a very thin chitosan layer.

## 2. Results and Discussion

**Figure 1:**
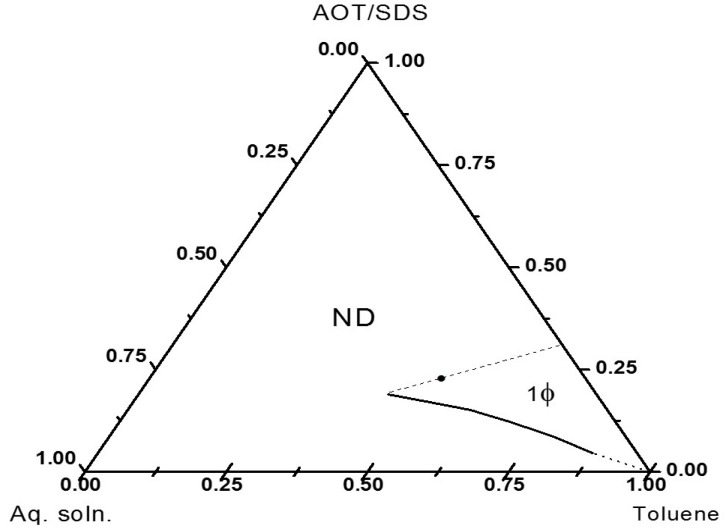
Phase diagram showing the microemulsion region (1ϕ). The microemulsion composition used in the precipitation reactions is shown by (●). The non-determined region (ND) is not relevant for this work.

The phase diagram at 80 °C of the system composed of 0.25 M aqueous solution of Fe chlorides, toluene and the AOT/SDS (2/1, w/w) mixture is depicted in Figure 1. This diagram shows a monophasic region which expands from the oil rich corner toward the central part.

The translucency and fluidity of the samples inside this area indicate that it corresponds to a microemulsion region. A microemulsion with a relatively high aqueous phase concentration (26 wt%) on the line of 30/70 weight ratio of surfactant/toluene (dot in the diagram) was chosen in order to increase the productivity of the precipitation reaction. The chosen position, in the central-right part of the diagram of the composition, suggests the corresponding microemulsion to be either a reverse or a bicontinuous one [25]. Measurements at 80 °C of the electrical conductivity of a sample with the composition pointed out above gave a very low value, 0.82 µS/cm, which is typical of a reverse microemulsion [26,27]. The poor electrical conductance of this type of microemulsions is due to the discontinuity of their nanostructures, since they are isolated swollen micelles dispersed in an oil continuous phase. In contrast, bicontinuous microemulsions show much better electrical conductance, typically on the order of 10^2^–10^3^µS/cm, as they consist of interconnected surfactant stabilized aqueous channels dispersed in the oil phase [28]. A reverse microemulsion with an aqueous phase content as high as 26 wt% is not common. The reason behind this unusual expansion of the reverse microemulsion region could be the well-known effect of increasing capacity for stabilization that ionic surfactants present as temperature increases.

**Figure 2:**
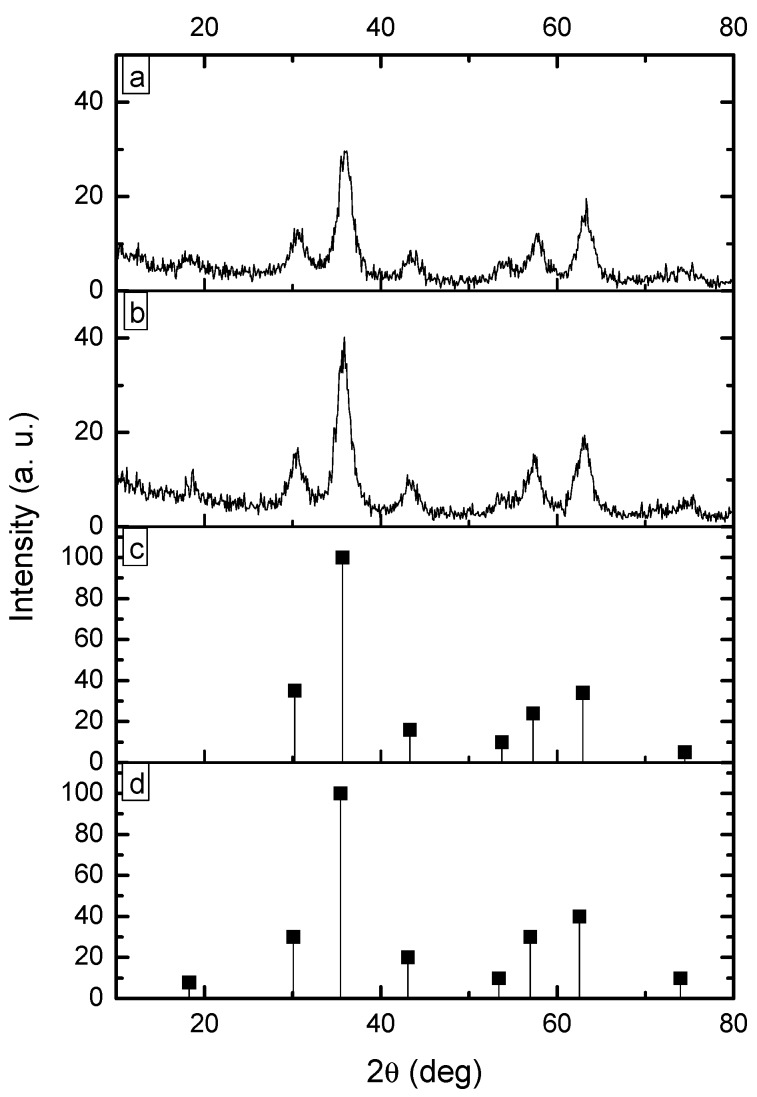
X-ray patterns of magnetic nanoparticles prepared by microemulsion precipitation: (**a**) without chitosan and (**b**) with chitosan. It is also included:(**c**) magnetite standard pattern and (**d**) maghemite standard pattern.

The precipitation reactions carried out without chitosan produced a black powder while those carried out in presence of chitosan gave rise to a brownish one. In both cases, the weight of dried product was close to 0.49 g. The X-ray diffraction pattern (XRDP) of the products obtained without chitosan (MH80) and with chitosan (MHQ80) are shown in Figure 2.

XRDP’s from the replicates (runs MH80-R and MHQ80-R) were not included; however they showed the same signal patterns. Figure 2 also includes the standard patterns of magnetite and maghemite, which were taken from the library of our X-ray equipment. As will be seen below, the results are in agreement with experiments showing that precipitation reactions from Fe^+2^ + Fe^+3^ aqueous solutions using aqueous ammonia usually render a mixture of magnetite and maghemite [29]. In accordance with Figure 2, XRDP’s of magnetite and maghemite display the same signals pattern in the range 30 to 75 2θ°. However, the maghemite pattern shows a signal at 18.22 2θ°, which is not displayed by that of magnetite. Meanwhile, XRDP’s from the products of runs MH80 and MHQ80 display the characteristic signals of magnetite and maghemite patterns in the range 30 to 75 2θ°, but they also show the 18.22 2θ° signal. Furthermore, as it is well-known, bulk maghemite displays a brown color while magnetite’s color is black [30]. From these data, it could be thought that the magnetic nanoparticles obtained would be composed of a magnetite-maghemite mixture or only maghemite. In the case of the products of MHQ80 and MHQ80-R precipitations, the brownish appearance of the samples would indicate a dominance of maghemite in the mixture of nanoparticles or an attenuation of the magnetite color due to the chitosan layer if the latter were dominant. In any case, the magnetic properties of the products would be only little affected, taking into account that the magnetization capability of maghemite is only slightly lower than that of magnetite [31].

The average crystallite size of the magnetic nanoparticles obtained in the precipitation reactions was determined from X-ray data using the well-known Scherrer equation:

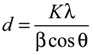
(1)


In this equation *d* is the average diameter of crystallite in nm; *K* is the dimensionless factor (0.9); λ is the X-ray wavelength (0.154 nm); β is the line broadening at half the maximum intensity in radians, and Ɵ is the Bragg’s angle. The *d* values estimated were 4.52 ± 0.01 and 4.67 ± 0.01 nm for particles from precipitations without and with chitosan, respectively. Despite this small difference in size, statistically it would appear that the presence of chitosan slightly promotes crystal growth. This result matches with that reported in our previous work [22] in which the particles prepared at 80 °C in a microemulsion containing 15 wt% 0.25 M aqueous solution of Fe chlorides in presence of chitosan registered sizes slightly larger than those obtained without this polymer.

**Figure 3:**
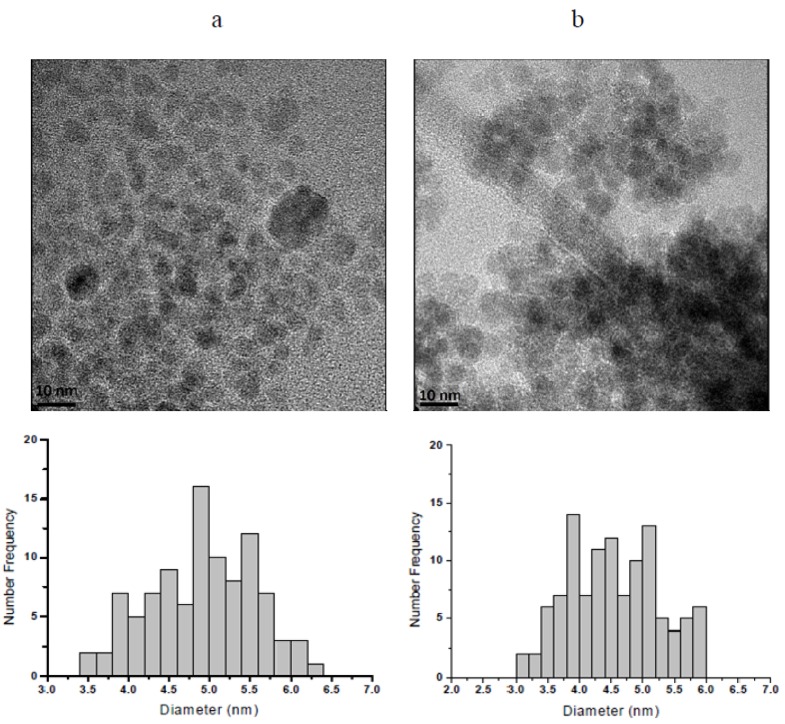
HRTEM micrographs of (**a**) naked magnetic nanoparticles (MH80) and (**b**) chitosan-coated magnetic nanoparticles (MHQ80). The corresponding histograms of particle sizes are also included.

The results obtained using the Scherrer equation show that the crystallite has average diameters ranging from 4.5 to 4.7 nm. However, the final size of magnetic nanoparticles does not necessarily correspond to this range, due to the possibility of crystallite aggregation leading to nanoparticles composed of two or more crystallites, which would rend nanoparticles with average diameters larger than those of crystallites. At this point in time, it is needed to mention that a crystallite is the smallest crystal that can be formed in a given process, that is, crystals with smaller size (on average) cannot be formed. Particle sizes determined by HRTEM are key to clarify the issue. HRTEM micrographs of nanoparticles from runs MH80 and MHQ80 along with the corresponding histograms of particle size are shown in Figure 3. The latter were elaborated by measuring the diameter of around 100 particles in the set of micrographs using an image analysis program (Image J 1.37c). From these data, *D_w_*, *D_n_* and PDI (*D_w_*/*D_n_*), being *D_w_* and *D_n_* the weight- and number-average diameters and PDI the polydispersity index, were calculated using the following equations:

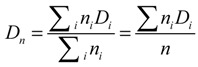
(2)

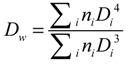
(3)


From the HRTEM measurements, values of 4.92 nm and 1.05 in *D_n_* and PDI, respectively were determined for nanoparticles prepared without chitosan. On the other hand, the corresponding values for MHQ80 were 4.54 nm and 1.07 while those for MHQ80-R were 4.55 nm and 1.08. The difference between the sizes of the two types of nanoparticles is very small, which, as was stated elsewhere [22], indicates that chitosan does not strongly affect the final nanoparticle size in the precipitation of magnetic nanoparticles in reverse microemulsions. Another result worth highlighting is the very low size distribution dispersity of the nanoparticles, typical of those prepared by precipitation in microemulsions [32]. An explanation for this result was provided when discussing a similar result obtained in our previous work [22]. On the other hand, the similarity between the values of crystallite diameters and those of nanoparticles determined by HRTEM leads to conclude that, in average, each magnetic nanoparticle is formed by one crystallite.

It has been shown thus far that the method used in this study allows the preparation of ultrafine magnetic nanoparticles in the presence of chitosan. The location of the chitosan on the surface of magnetic nanoparticles will be demonstrated in what follows.

**Figure 4:**
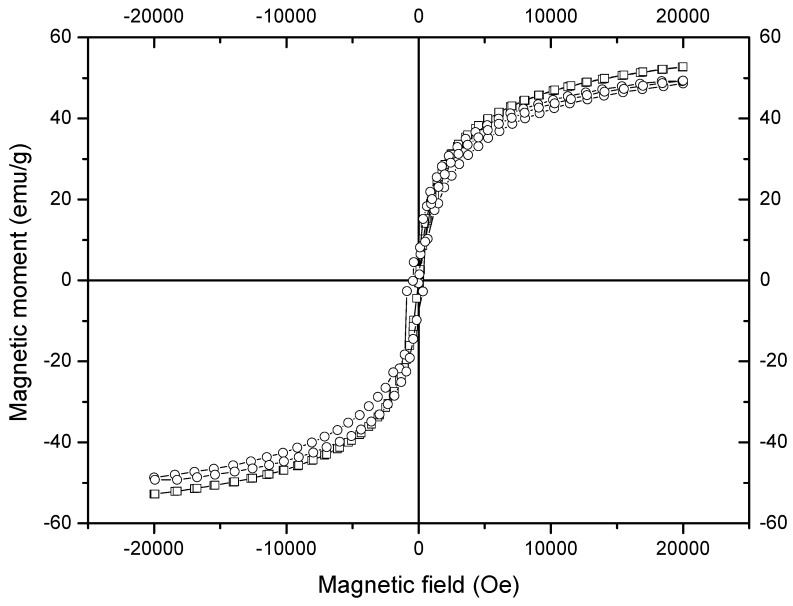
Magnetization curves at room temperature of (□) naked magnetic nanoparticles (MH80) and (○) chitosan-coated magnetic nanoparticles (MHQ80).

The results of magnetic characterization at room temperature are shown in Figure 4 and Table 1. The magnetization curves represented in this figure correspond to the nanoparticles from MH80 and MHQ80. The replicates (not shown) display similar behavior to those of the corresponding original run. From the form of the magnetization curves in Figure 4 is evident that the magnetic nanoparticles prepared in this study do not attain magnetic saturation at the highest magnetic field applied (20 kOe); a similar behavior was observed in our previous works [17,18,22], in which an explanation based on the high significance of the surface atoms of the nanoparticles was provided [22]. The nanoparticles prepared without chitosan attained higher magnetization values than those shown by the nanoparticles obtained in presence of the polymer; this difference was taken to indicate the chitosan attachment to the magnetic nanoparticles. On the other hand, the magnetization at the highest magnetic field attained by the nanoparticles prepared in presence of chitosan in this study (49.0 ± 0.7 emu/g) is significant. Assuming, as it will be further demonstrated, that chitosan is attached to the magnetic nanoparticles, the obtained coated magnetic nanoparticles show magnetization values slightly lower than those of the CMNPs prepared by our group in a microemulsion with lower aqueous phase content (52.9 emu/g) [22]. However, the magnetization results obtained by our group are higher than those reported by other groups for larger CMNPs: 36 [4], 35 [15], 35 [16] and 13.4 emu/g [19] and also higher than that of CMNPs of similar size: 25 emu/g [11]. We ascribe the differences to arise, in part, from the lower chitosan content in our particles and to the fact that the precipitation reactions were carried out at relatively high temperature (80 °C), which is well-known to promote higher crystallinity, leading to higher magnetization values [33]. Furthermore, the coercivity and remnant magnetization values displayed by both types of nanoparticles demonstrate their superparamagnetic behavior [30].

**Table 1:** Results from magnetic measurementsof nanoparticles.

Run	Final Magnetization (emu/g)	Remnant Magnetization (emu/g)	Coercivity(Oe)
MH80	52.8	0.1	3.0
MH80-R	51.6	0.1	2.5
MHQ80	49.3	0.3	39.0
MHQ80-R	49.7	0.5	17.5

**Figure 5:**
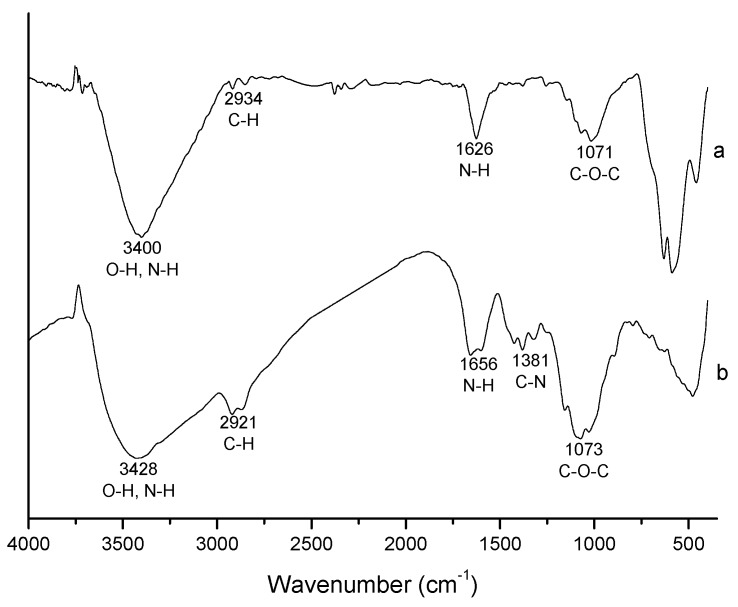
FTIR spectrum of chitosan-coated magnetic nanoparticles (MHQ80) prepared by microemulsion precipitation (**a**). Chitosan spectrum is also included (**b**).

The FTIR spectra of magnetic nanoparticles prepared in presence of chitosan (MHQ80) and that of pure chitosan are shown in Figure 5. The former displays four of the five typical characteristic absorption bands of pure chitosan in accordance with the literature [34,35]: 3,428 (O-H and N-H stretching vibrations), 2,921 (C-H stretching vibrations), 1,656 (N-H bending vibrations) and 1,073 cm^−1^ (C-O-C stretching vibrations). The bands at 1,320–1,420 cm^−1^ (C-N stretching vibrations) sharply decrease in the spectrum of magnetic nanoparticles prepared in the presence of chitosan. The spectrum of MHQ80-R (not shown) is similar to that of MHQ80. Taking into account that a chitosan layer on magnetic nanoparticles is expected, this band decrease could be ascribed to the formation of N-Fe complex. Moreover, given that the products of the precipitation reactions in presence of chitosan were exhaustively washed and magnetically recovered, only chitosan attached to the magnetic nanoparticles would appear in the final product. Thus, it can be concluded that all the chitosan in the final product is coating the surface of magnetic nanoparticles. Nevertheless, it is possible that some magnetic nanoparticles remained uncoated.

The characterization results of the products of precipitation reaction indicate the obtaining of magnetic nanoparticles coated by chitosan. To quantify the fraction of this polymer as well as the remaining surfactant in the final product, carbon and sulfur contents in the products from precipitation reactions MHQ80 and MHQ80-R were determined. The results of these determinations were 2.84% and 2.66% carbon content, respectively, while the sulfur contents were smaller than 0.005% in both products. The carbon content in the products along a calculated value of 45.23% for carbon content in a repetitive unit of chitosan with a 75% deacetylation degree, were used to estimate chitosan contents of 6.27% and 5.88% for MHQ80 and MHQ80-R, respectively. The very low sulfur content in the products indicates that the surfactant content in the coated magnetic nanoparticles was negligible, concluding that the coated magnetic nanoparticles were composed only of chitosan and magnetic material. The results above allowed to calculate 66.9 and 62.5 mg chitosan covering each gram of magnetic material, for MHQ80 and MHQ80-R, respectively, which are higher than the theoretical value (≈53.0 mg/g) assuming 100% conversion to magnetite in the precipitation reaction and all chitosan used in the recipe covering the nanoparticles. This apparent contradiction could arise from conversions slightly lower than 100% in the precipitation reactions along the formation of a mixture of magnetite and maghemite instead of only magnetite. These results indicate that the known mechanism in the preparing of magnetic nanoparticles in reverse microemulsions is not modified by the presence of chitosan. A possible mechanism of formation of coated magnetic nanoparticles in this media was already proposed by our group [22].

An important feature of CMNPs is the number of the amino groups on their surface. An estimation of the number of these groups per each coated magnetic nanoparticle was obtained as follows. Using the *D_n_* value of the coated magnetic nanoparticles, the contents of magnetic material and chitosan along the density values of 5.2 g/mL for magnetite [36] and 1.34 g/mL for chitosan [37], a chitosan layer thickness and then the number of nanoparticles per one gram of CMNP was calculated. These results along the amino groups concentration in one gram of the CMNP, measured by the ninhydrin method, allowed us to calculate the number of amino groups per each coated magnetic nanoparticle. Data from the recipe of precipitation reactions MHQ80 and MHQ80-R were used in the calculation sequence. The results obtained by the calculation sequence above described are shown in Table 2.

**Table 2:** Surface features and nanoparticle number per mass unit of the chitosan-coated magnetic nanoparticles.

Run	Chitosan Layer Thickness(nm)	Nanoparticles per Gram of CMNP × 10^−18^	NH_2_ (mmoles per gram of CMNP) ^1^	NH_2_ (groups per particle)
MHQ80	0.17	4.1	0.1422	20.3
MHQ80-R	0.16	4.1	0.1251	17.8

^1^ Determined by the ninhydrin method.

Data in Table 2 indicate that the chitosan layer on the magnetic nanoparticles is very thin(≈ 0.16–0.17 nm), similar to that calculated for CMNPs whose preparation by precipitation in reverse microemulsions was previously reported by our group [22]. Assuming an homogeneous distribution of the polymer on the surface of the nanoparticles, the value almost corresponds to a molecular monolayer. On the other hand, the number of amino groups per each coated magnetic nanoparticle, around 20 and 17 for MHQ80 and MHQ80-R, respectively, should be noted. These values are slightly higher than those obtained for CMNP previously prepared [22], however they are much lower than the value of 135 previously obtained by our group when the one-step coprecipitation method was used to manufacture chitosan-coated magnetic nanoparticles [18].

The difference between the number of amino groups on the surface of CMNPs reported in this study and that of those obtained by one-step coprecipitation method is noticeable taking into account that the chitosan content in both CMNPs was very similar (≈ 5%–6%). The smaller particle diameter of CMNPs prepared by microemulsion, 4.5 nm, compared to the corresponding value, 10 nm, obtained by the one-step coprecipitation method, would not be the only cause for the observed difference, since the size reduction factor was close to 2.2, which leads to an area per particle reduction factor of 4.8, and considering an equal number of amino groups per area unit, the nanoparticles obtained by microemulsion precipitation should contain nearly 28 amino groups per particle. This result can be explained as a consequence of forming chitosan aggregates instead of a homogeneous layer on the nanoparticles surface.

The tests for Pb^2+^ removal from a Pb(NO_3_)_2_ aqueous solution by the nanoparticles from MHQ80 and MHQ80-R precipitation reactions are based on the ability of chitosan to chelate heavy metal ions through its amino groups. The behavior of both samples are very similar, since after only 10 min, 98.2% of the Pb^2+^ ions were chelated by the CMNP from MHQ80 reaction, while Pb^2+^ ions were undetectable in the case of those of MHQ80-R. These results show the high capacity of the CMNP prepared in this study to chelate heavy metal ions through their surface amino groups provided by chitosan.

Data in Table 2 and the amounts of CMNPs used in the Pb^2+^ removal runs allowed us to determine that the amino groups on the surface of all dispersed nanoparticles were 5.1 × 10^18^ and 4.5 × 10^18^ for MHQ80 and MHQ80-R nanoparticles, respectively. Since the Pb^2+^ions content in the initial solution is 1.22 × 10^18^, the ratio of amino groups to chelated Pb^2+^ ions is approximately 4 for both MHQ80 and MHQ80-R nanoparticles. In our prior work, where CMNPs were prepared by a one-step coprecipitation method [18], that ratio was found to be 3.4, that is, slightly lower. However, in that instance, only around 70% of initial Pb^2+^ ions were chelated at the end of the run (50 min). The greater efficacy showed by CMNPs obtained via microemulsion could arise from the difference in the total amino groups on the surface of the dispersed nanoparticles. As noted above, the nanoparticles from microemulsion have values of 4.5–5.1 × 10^18^, while those prepared via coprecipitation show a value of 3.2 × 10^18^. Finally, an important point to note here is the productivity obtained in this study. Since reverse microemulsions usually contain low aqueous phase concentrations (≤ 15 wt%) [26], their precipitation productivities are low. In fact, 0.28 g CMNP/100 g microemulsion was the productivity obtained in our previous work on preparation of CMNPs by precipitation in reverse microemulsion containing 15 wt% aqueous phase [22]. In the present report, a reverse microemulsion containing an unusually high aqueous phase concentration (26 wt%) was employed, allowing us to obtain 0.49 g CMNP/100 g microemulsion, which constitutes an important increase in the productivity with respect to that achieved in our previous work.

## 3. Experimental

### 3.1. Reagents

Ferric chloride hexahydrate (FeCl_3_·6H_2_O, 99%), ferrous chloride tetrahydrate (FeCl_2_·4H_2_O, 98%), aqueous ammonia (NH_4_OH, 57.6 wt%), sodium dodecyl sulfate (SDS, 98%), sodium bis(2-ethylhexyl) sulfosuccinate (AOT, 98%), ninhydrin (97%) and chitosan with low molecular weight and 75% deacetylation degree, all of them from Aldrich (Toluca, Edo. Méx., México) were used as received. Lead nitrate, Pb(NO_3_)_2_, 99.7%, from J.T. Baker (Monterrey, N.L., México), was also used as received. De-ionized and triple-distilled water was drawn from a Millipore system.

### 3.2. Phase Diagram Determination

The determination of the microemulsion region at 80 °C was carried out by titration with 0.25 M aqueous solution of a FeCl_3_·6H_2_O/FeCl_2_·4H_2_O mixture (3/2, mol/mol) of solutions of surfactants (AOT/SDS, 2/1, w/w)/toluene at the following weight ratios: 5/95, 10/90, 15/85, 20/80, 25/75 and 30/70. The transparency or translucency appearance of the samples indicates that their composition correspond to a microemulsion. The extension of the microemulsion region was detected visually at each one of the constant (AOT/SDS)/toluene lines studied, when the appearance of the samples turned opalescent. Then, samples with compositions slightly below and above that of the visually determined phase boundary were prepared by weighting each component and allowing to reach equilibrium in a water bath at 80 °C to determine more precisely the phase boundary.

### 3.3. Preparation of Magnetic Nanoparticles

The precipitation reactions, each one in duplicate, were carried out in a 150 mL jacketed glass reactor equipped with a reflux condenser an inlet for aqueous ammonia feed and a mechanical agitator operated at 300 rpm. The procedure started forming a microemulsion (100 g) by mixing at 80 °C, 22.2 wt% (AOT/SDS, 2/1, w/w), 51.8 wt% toluene and 26.0 wt% 0.25 M aqueous solution of a FeCl_3_·6H_2_O/FeCl_2_·4H_2_O mixture (3/2, mol/mol) containing 0.1 wt% chitosan. Precipitation reactions without chitosan were also carried out. To initiate the precipitation, a shot of 3.9 g aqueous ammonia was added to the reactor allowing the reaction proceed for 30 min. At the end of the reaction, the particles were recovered by using a permanent magnet, washed at least 10 times with water-acetone (81/19, w/w) and then dried.

### 3.4. Pb^2+^ Removal Test

As it is well-known, the amino groups of chitosan have the capability to chelate heavy metal ions. To probe the capability of the chitosan-coated magnetic nanoparticles as a material to remove this kind of ions, 50 mL of a Pb(NO_3_)_2_ aqueous solution containing 10 ppm of Pb^2+^ was prepared. Then 60 mg dried CMNPs were added to the solution followed by ultrasonication for 50 min at room temperature taking samples during the process each 10 min. The chitosan-coated magnetic nanoparticles with the chelated Pb^2+^ ions were recovered by using a permanent magnet. After several water washing, the particles were dried and the concentration of Pb^2+^ in the samples was measured by atomic absorption spectroscopy on a Varian(México, D.F., México)SpectrAA 220 instrument.

### 3.5. Characterization

Measurements of electrical conductivity of microemulsions were carried out at 80 °C and 1 KHz with a Hachsension 5 conductivity meter. A Siemens (México, D.F., México) D-5000 diffractometer using Cu-K_α_ (λ = 1.5418 Å) as incident radiation was utilized for X-ray analysis of the products. The size and morphology of the particles were determined in a high-resolution transmission electron microscope (HRTEM) Titan-300 kV for which samples were prepared by dispersing the resulting powders in water with ultrasonication and then depositing the dispersion on a copper grid. The magnetic characterization of the nanoparticles was performed using a model 6000 Physical Properties Measurement System from Quantum Design (México, D.F., México), in vibrating sample magnetometer (VSM) mode, with an applied field between −20.0 to 20.0 kOe at room temperature. An Eltra (Saltillo, Coah., México) CS800 induction furnace was employed to determine the carbon and sulfur content in the nanoparticles by the combustion method. Fourier transform infrared spectrometry (FTIR) was carried out in a Magna IR 550 from Nicolet (México, D.F., México) with germanium crystal. For this, the dried particles sample was ground with potassium bromide powder and subsequently pressed to form a disk, which was analyzed in the apparatus.

### 3.6. Amino Groups Determination

The ninhydrin method was applied to determine the amino groups on the surface of CMNP [38]. Glycine was used as standard to construct the calibration curve in the range 1 to 5 mM. To make the measurements, 0.1 g of CMNP were dispersed in 1 mL of water and then 0.6 mL of ninhydrin reagent were added. After that, the dispersion was boiled for 30 min. The amino groups concentration in aqueous phase of the dispersion was determined by readings of absorbance at 570 nm in a Cintra (México, D.F., México) 20 Double Beam UV-Vis spectrophotometer.

## 4. Conclusions

Chitosan-coated magnetic nanoparticles were prepared in one-step by precipitation in a reverse microemulsion containing an unusual high-aqueous phase concentration. The average diameters of the crystallites (4.5 nm) were similar to those of coated nanoparticles, which suggests the absence of nanoparticle aggregation and that on average each nanoparticle is composed of one crystallite. As a consequence of chitosan only constituting a very thin layer, the magnetization of the coated nanoparticles was only slightly lower than that shown by the naked magnetic nanoparticles. However the former attained magnetization values close to 49 emu/g, which are relatively high, taking into account the very small size of the nanoparticles. It is believed that this effect is due to the higher crystallinity promoted by the high temperature at which the precipitation reactions were carried out. The superparamagnetism displayed by the chitosan-coated nanoparticles reported, their high efficacy in recovering Pb^2+^ ions in aqueous solution as well as the relatively high productivity of the process used in their preparation, open the possibility for their use in applications such as water contamination removal, diagnosis and sensors manufacture, to mention a few.

## References

[1] Guimaraes A.P. (2009). Principles of Nanomagnetism.

[2] Varadan V.K., Chen L., Xie J. (2008). Nanomedicine: Design and Applications of Magnetic Nanomaterials, Nanosensors and Nanoystems.

[3] Woo K., Hong J., Choi S., Lee H.W., Ahn J.P., Kim C.S., Lee S.W. (2004). Easy synthesis and magnetic properties of iron oxide nanoparticles. Chem. Mater..

[4] Yuwei C., Jianlong W. (2011). Preparation and characterization of magnetic chitosan nanoparticles and its application for Cu(II) removal. Chem. Eng. J..

[5] Tang S.C.N., Lo I.M.C. (2013). Magnetic nanoparticles: Essential factors for sustainable environmental applications. Water Res..

[6] Cassidy P.J., Radda G.K. (2005). Molecular imaging perspectives. J. R. Soc. Interface.

[7] Bertorelle F., Wilhelm C., Roger J., Gazeau F., Ménager C., Cabuil V. (2006). Fluorescence-modified superparamagnetic nanoparticles: Intracellular uptake and use in cellular imaging. Langmuir.

[8] Xu C., Sun S. (2009). Superparamagnetic nanoparticles as targeted probes for diagnostic and therapeutic applications. Dalton Trans..

[9] Qu J., Liu G., Wanga Y., Hong R. (2010). Preparation of Fe_3_O_4_-chitosan nanoparticles used for hyperthermia. Adv. Powder Technol..

[10] Marinescu G., Patron L., Culita D.C., Neagoe C., Lepadatu C.I., Balint I., Bessais L., Cizmas C.B. (2006). Synthesis of magnetite nanoparticles in the presence of aminoacids. J. Nanopart. Res..

[11] Unsoy G., Yalcin S., Khodadust R., Gunduz G., Gunduz U. (2012). Synthesis, optimization and characterization of chitosan-coated iron oxide nanoparticles produced for biomedical applications. J. Nanopart. Res..

[12] Kurita K. (2001). Controlled functionalization of the polysaccharide chitin. Prog. Polym. Sci..

[13] Dung D.T.K., Hai T.H., Phuc L.H., Long B.D., Vihn L.K., Truc P.N. (2009). Preparation and characterization of magnetic nanoparticles with chitosan coating. J. Phys. Conf. Ser..

[14] Pan C., Hu B., Li W., Sun Y., Ye H., Zeng X. (2009). Novel and efficient method for immobilization and stabilization of *B*-d-galactosidase by covalent attachment onto magnetic Fe_3_O_4_-chitosan nanoparticles. J. Mol. Catal. B-Enzym..

[15] Hong S., Rhee I. (2007). Relaxivity of hydrogen protons of water molecules in the aqueous solutions of dextran- and chitosan-coated ferrite nanoparticles. Int. J. Magn. Reson. Imag..

[16] Wu Y., Wang Y., Luo G., Dai Y. (2009). In situ preparation of magnetic Fe_3_O_4_-chitosan nanoparticles for lipase immobilization by cross-linking and oxidation in aqueous solution. Bioresour. Technol..

[17] Gregorio-Jáuregui K.M., Pineda M.G., Rivera-Salinas J.E., Hurtado G., Saade H., Martínez J.L., Ilyina A., López R.G. (2012). One-step method for preparation of magnetic nanoparticles coated with chitosan. J. Nanomater..

[18] Osuna Y., Gregorio-Jauregui K.M., Gaona-Lozano J.G., de la Garza-Rodríguez I.M., Ilyna A., Díaz Barriga-Castro E., Saade H., López R.G. (2012). Chitosan-coated magnetic nanoparticles with low chitosan content prepared in one step. J. Nanomater..

[19] Liu Y., Jia S., Wu Q., Ran J., Zhang W., Wu S. (2011). Studies of Fe_3_O_4_-chitosan nanoparticles prepared by co-precipitation under the magnetic field for lipase immobilization. Catal. Commun..

[20] Wang Y., Li B., Xu F., Jia D., Feng Y., Zhou Y. (2012). *In vitro* cell uptake of biocompatible magnetite/chitosan nanoparticles with high magnetization: A single step synthesis approach for in-situ-modified magnetite by amino groups of chitosan. J. Biomater. Sci. Polym. Ed..

[21] Li W., Xiao L., Qin C. (2011). The characterization and thermal investigation of chitosan-Fe_3_O_4_ nanoparticles synthesized via a novel one-step modifying process. J. Macromol. Sci. Part A: Pure Appl. Chem..

[22] López R.G., Pineda M.G., Hurtado G., Díaz de León R., Fernández S., Saade H., Bueno D. (2013). Chitosan-coated magnetic nanoparticles prepared in one step by reverse microemulsion precipitation. Int. J. Mol. Sci..

[23] Lindman B., Friberg S.E., Kumar P., Mittal K.L. (1999). Handbook of Microemulsion Science and Technology.

[24] Osseo-Asare K., Kumar P., Mittal K.L. (1999). Handbook of Microemulsion Science and Technology.

[25] Salager J.L., Anton R.E., Kumar P., Mittal K.L. (1999). Handbook of Microemulsion Science and Technology.

[26] Eicke H.F., Borkovec M., Gupta B.D. (1989). Conductivity of water-in-oil-microemulsions: A quantitative charge fluctuation model. J. Phys. Chem..

[27] Borkovec M., Eicke H.F., Hammerich H., Gupta B.D. (1988). Two percolation processes in microemulsions. J. Phys. Chem..

[28] Ezrahi S., Aserin A., Garti N., Kumar P., Mittal K.L. (1999). Handbook of Microemulsion Science and Technology.

[29] Loo A.L., Pineda M.G., Saade H., Treviño M.E., López R.G. (2008). Synthesis of magnetic nanoparticles in bicontinuous microemulsions. Effect of surfactant concentration. J. Mater. Sci..

[30] Cornell R.M., Schwertmann U. (1996). The Iron Oxides: Structure, Properties, Reactions, Occurrence and Uses.

[31] Demortiere A., Panissod P., Pichon B.P., Pourroy G., Guillon D., Donnio B., Bégin-Colin S. (2011). Size-dependent properties of magnetic iron oxide nanocrystals. Nanoscale.

[32] Berry C.C. (2009). Progress in functionalization of magnetic nanoparticles for applications in biomedicine. J. Phys. D: Appl. Phys..

[33] Qi H., Ye J., Tao N., Wen M., Chen Q. (2009). Synthesis of octahedral magnetite microcrystals with high crystallinity and low coercive field. J. Cryst. Growth.

[34] Guo L., Liu G., Hong R.Y., Li H.Z. (2010). Preparation and characterization of chitosan poly(acrylic acid) magnetic microspheres. Mar. Drugs.

[35] Chen L., Tang C., Ning N., Wan C., Fu Q., Zhang Q. (2009). Preparation and properties of chitosan/lignin composite films. Chin. J. Polym. Sci..

[36] Liley P.E., Reid R.C., Buck E., Perry R.H., Green D.W., Maloney J.O. (1984). Physical and Chemical Data, in Chemical Engineering Handbook.

[37] Hsieh W.C., Chang C.P., Lin S.M. (2007). Morphology and characterization of 3D micro-porous structured chitosan scaffolds for tissue engineering. Colloids Surf. B: Biointerfaces.

[38] Kaiser E., Colescott R.L., Bossinger C.D., Cook P.I. (1970). Color test for detection of free terminal amino groups in the solid-phase synthesis of peptides. Anal. Biochem..

